# CTDB: An Integrated Chickpea Transcriptome Database for Functional and Applied Genomics

**DOI:** 10.1371/journal.pone.0136880

**Published:** 2015-08-31

**Authors:** Mohit Verma, Vinay Kumar, Ravi K. Patel, Rohini Garg, Mukesh Jain

**Affiliations:** Functional and Applied Genomics Laboratory, National Institute of Plant Genome Research (NIPGR), New Delhi, India; University of Toronto, CANADA

## Abstract

Chickpea is an important grain legume used as a rich source of protein in human diet. The narrow genetic diversity and limited availability of genomic resources are the major constraints in implementing breeding strategies and biotechnological interventions for genetic enhancement of chickpea. We developed an integrated Chickpea Transcriptome Database (CTDB), which provides the comprehensive web interface for visualization and easy retrieval of transcriptome data in chickpea. The database features many tools for similarity search, functional annotation (putative function, PFAM domain and gene ontology) search and comparative gene expression analysis. The current release of CTDB (v2.0) hosts transcriptome datasets with high quality functional annotation from cultivated (desi and kabuli types) and wild chickpea. A catalog of transcription factor families and their expression profiles in chickpea are available in the database. The gene expression data have been integrated to study the expression profiles of chickpea transcripts in major tissues/organs and various stages of flower development. The utilities, such as similarity search, ortholog identification and comparative gene expression have also been implemented in the database to facilitate comparative genomic studies among different legumes and Arabidopsis. Furthermore, the CTDB represents a resource for the discovery of functional molecular markers (microsatellites and single nucleotide polymorphisms) between different chickpea types. We anticipate that integrated information content of this database will accelerate the functional and applied genomic research for improvement of chickpea. The CTDB web service is freely available at http://nipgr.res.in/ctdb.html.

## Introduction

Chickpea is an agriculturally important legume crop, which ranks second in cultivated area and third in production among legumes worldwide. It is a good source of proteins and dietary fibers in human diet. Similar to other members of leguminosae family, chickpea can restore the soil fertility by fixing atmospheric nitrogen. India ranks first in production as well as consumption of chickpea (FAOSTAT, 2012; http://faostat.fao.org). The chickpea productivity is substantially low worldwide and affected by several biotic and abiotic factors. Hence, the development of high-yielding chickpea varieties is a pressing need in order to meet demands of an overgrowing population. However, due to limited availability of genomic and genetic resources, the progress in the improvement of chickpea yield has been limited in the past years.

Over the past three decades, extensive research has been carried out for the chickpea improvement. However, the narrow genetic diversity in cultivated and wild accessions restricts the genetic improvement by limiting introduction of diverse germplasm using conventional/molecular-breeding approaches. The cultivated chickpeas are categorized into kabuli and desi types based on phenotypic appearance of the seed (size, shape and color). Wild chickpea (*Cicer reticulatum*) is the progenitor of domesticated chickpea (*C*. *arietinum*) and can serve as a potential source of genes responsible for several agronomically important traits [1]. The availability of next generation sequencing technologies has catalyzed the genomics research in chickpea. The draft genome sequences of kabuli (CDC frontier) and desi (ICC4958) chickpea genotypes have been released [2,3]. The transcriptome datasets of wild, kabuli and desi type chickpea have been analyzed to provide resources for gene discovery and development of functional molecular markers [4–9]. Furthermore, gene expression studies from different tissues/organs and/or environmental conditions have also been performed to reveal the putative functions of chickpea transcripts [4,6,10,11]. Recently, two microsatellite databases, CicArMiSatDB [12] and CMsDB (http://nipgr.res.in/CMsDB.html), have been developed to identify functional microsatellite markers in the chickpea genome for various applications. The availability of these high-throughput data has opened several avenues for genetics and genomics research in chickpea. In addition, a few web resources, such as SoySeq, SoyPlex, MtGEA, LjGEA and LegumeIP, are available for functional and/or comparative genomics in other legumes [13–18]. Most of these web resources/databases host mainly gene expression profile datasets.

Despite the extensive research in the development of genomics resources for chickpea, it is still difficult for a biologist to mine these data manually to parse out the relevant biological information. To make the information retrieval easy, we developed the Chickpea Transcriptome Database (CTDB) as an integrated web resource to provide comprehensive information on various aspects of transcriptome data from different (desi, kabuli and wild) chickpea genotypes. The database provides several user-friendly utilities for similarity search, functional annotation and gene expression data from different tissues/organs in chickpea and an option to conduct comparative genomics studies with/among different legumes. Furthermore, the database provides access to the functional molecular markers (microsatellites and single nucleotide polymorphisms) in the transcriptomes of different chickpea types. We envisage that the CTDB would serve as a potential resource for functional and applied genomics research in chickpea.

## Results and Discussion

### Database overview

**Fig 1:**
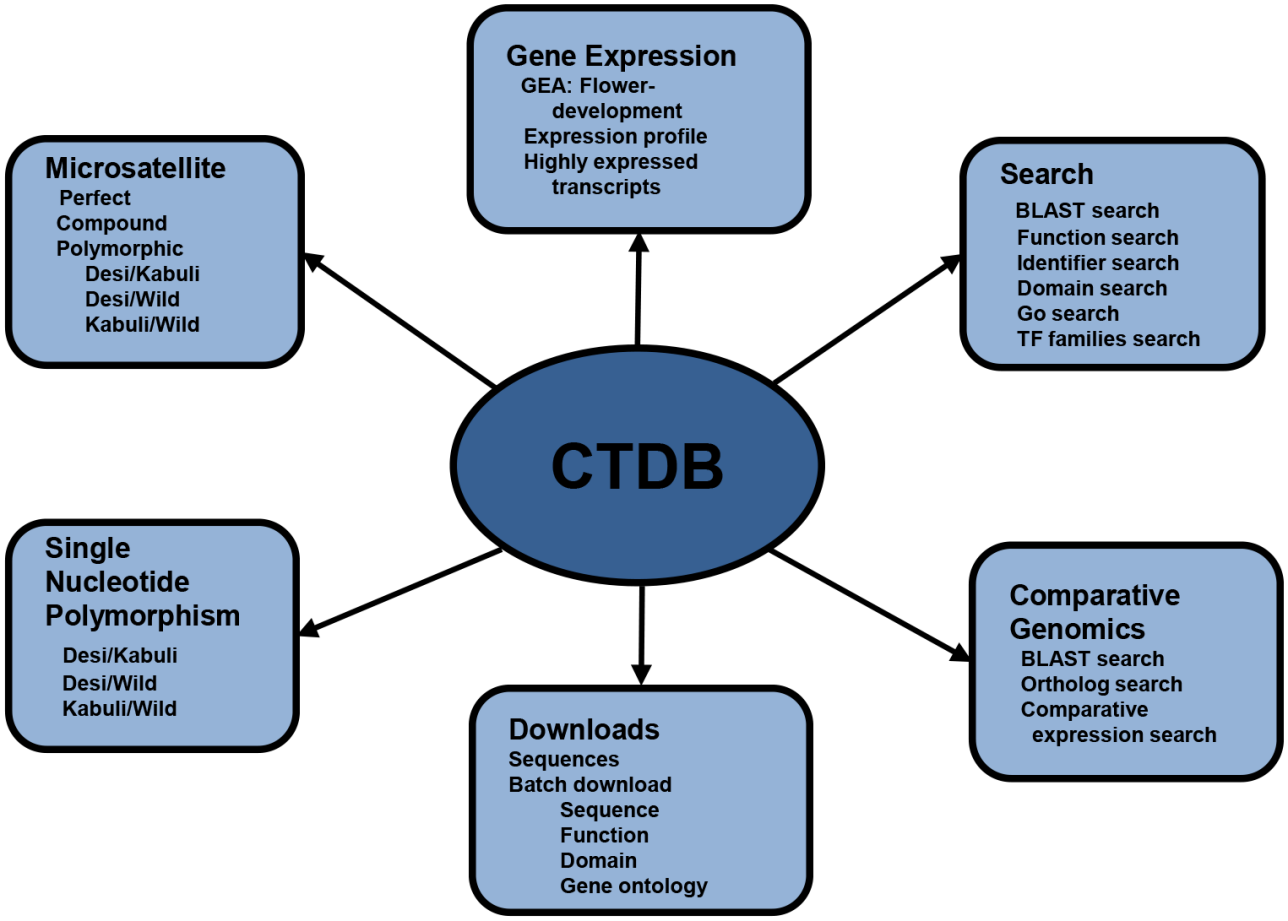
The complete schema of the CTDB (v2.0) database. Different modules with various utilities integrated in the database have been highlighted.

**Fig 2:**
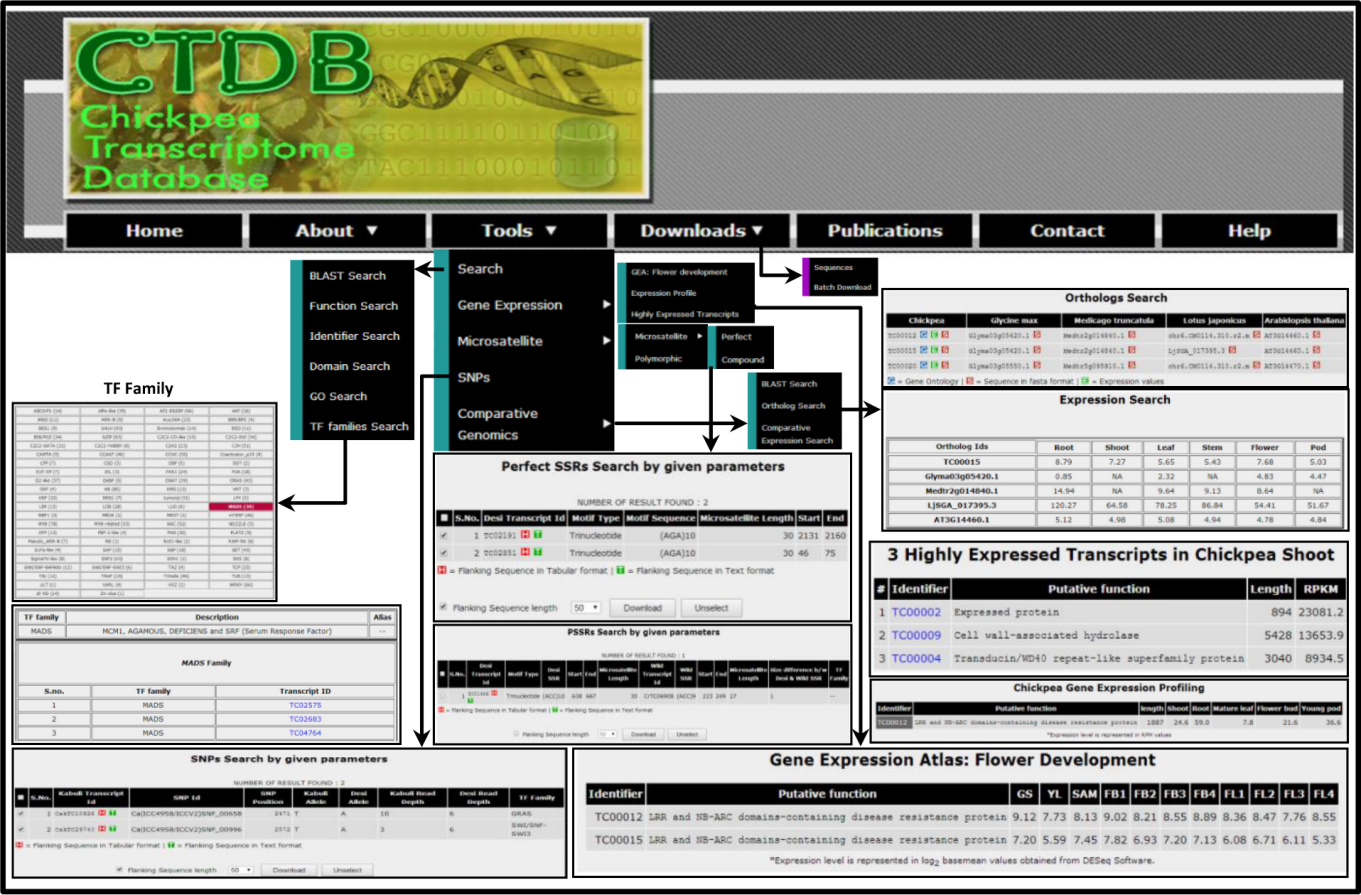
Screenshots of the CTDB (v2.0) for searching, exploring and visualization for different queries. The new/updated features/utilities, including TF family search, GEA: Flower development, Microsatellite, SNPs and Comparative Genomics are illustrated.

In view of establishment of an integrated platform for comprehensive transcriptome analysis in chickpea, we have developed CTDB to provide public access to various aspects of transcriptome for wild, desi and kabuli chickpea genotypes [5, 7–8]. The CTDB has a user-friendly interface for the efficient retrieval and visualization of information of interest with the aid of multiple search options. The data retrieval system of CTDB has been hierarchically organized into five major sections (Search, Gene Expression, Microsatellite, SNPs and Comparative Genomics), with several utilities as shown in Fig 1. Using these options, users can sail through the database and visualize/browse and download various functional (gene annotation and TF families), behavioral (gene expression), and structural (microsatellite and SNP) features of the chickpea transcriptome (Fig 2). Further, CTDB allows comparative analysis of chickpea transcriptome with other legumes (*Glycine max*, *Medicago truncatula* and *Lotus japonicus*) and model plant Arabidopsis.

### Key features of the CTDB

#### Similarity and function search

The database provides various utilities under “Search” menu for data retrieval, similarity search and functional annotation of query transcript sequence(s).

- BLAST search allows user to find homologous transcript or protein sequences for the sequence(s) of interest in the selected chickpea genotype.
- Function search utility facilitates the retrieval of chickpea transcripts for a given keyword query, for instance proteases or RNA polymerases.
- The identifier search enables users to retrieve the putative function and gene ontology (GO) term(s) associated with a given transcript.
- Domain search offers the identification of a set of transcripts harboring the Pfam domain of interest. The interface also allows users to access the putative function, gene ontology (GO) and sequences in fasta format.
- GO search utility provides an option to explore the GO associated with a given transcript. Users can use either the transcript identifier as a query to retrieve the GO terms associated with the transcript or the GO identifier to list all transcripts linked to a GO term.
- Transcription Factor (TF) family search provides access to various transcription factor families in chickpea. Each TF family links to a list of members and their expression profiles.

These search utilities can help to quickly query the chickpea transcriptome data for numerous functional genomics related applications.

#### Gene expression

The gene expression analysis can provide insights into the putative function of individual genes. Such analysis for a set of tissues/developmental stages has been performed in chickpea [4,11]. The CTDB offers three options for the gene expression search in chickpea; “GEA (gene expression atlas): flower development”, “Expression Profile” and “Highly Expressed Transcripts”.

The “GEA: Flower Development” option presents a resource to analyze the expression of gene(s)/transcript(s) during different developmental stages, including germinating seedling, young leaf, shoot apical meristem and eight stages of flower development (early flower bud to senescing flower) in chickpea [11]. Single or multiple transcript identifiers can be queried to study their expression profile(s) (Fig 2). The second gene expression analysis option, “Expression Profile”, provides expression information in major tissue samples (shoot, root, mature leaf, flower bud and young pod) for single/multiple transcript(s). Third option of “Highly Expressed Transcripts” has been provided to enlist most abundant transcripts in a particular tissue of chickpea. A hyperlink to the expression profile of each transcript has also been provided in the output. Overall, CTDB offers an opportunity to systematically identify expression patterns of individual or a set of transcripts in different tissues of chickpea. This tool provides an opportunity to the user(s) to choose appropriate gene(s) for functional analysis via reverse genetics approaches. The expression data provided in the database have been used in previous studies for characterization of individual gene [19] and gene family [20], which demonstrate the confidence of scientific community in our database.

#### Microsatellite search

Microsatellites (simple sequence repeats, SSRs) are important molecular markers for the study of genetic variations among different cultivars using marker-assisted selection and mapping of quantitative trait loci [21–23]. The screening of polymorphic SSR markers using transcriptomes is one of the approach to investigate potential functional markers in chickpea [4, 8]. The CTDB provides an opportunity for the screening of microsatellites along with their physical location on the transcript(s) in different (desi, kabuli and wild) chickpea genotypes (Fig 2). The database provides separate retrieval systems for different microsatellite repeat types, including perfect, compound and polymorphic microsatellites. The interface for the perfect microsatellite search provides various parameters, such as chickpea genotype, repeat type, repeat sequence, repeat number, and length and position of the microsatellite(s). The compound microsatellite is a combination of more than one type of perfect repeats, which makes their search challenging. The CTDB offers an efficient query builder to search the compound microsatellites using different parameters, including repeat type, repeat sequence and number of repeat units. In addition, CTDB provides an access to the polymorphic SSRs between different chickpea genotypes. The database hosts a total of 130, 561 and 493 polymorphic SSRs between kabuli/desi, desi/wild and kabuli/wild, respectively. The user interface for all types of microsatellites offers an option to download downstream and upstream flanking sequences to the microsatellite, which can be used for designing PCR primers for genotyping applications. The utility of these data to discover the microsatellites for construction of the marker-trait linkages and the identification of genes associated with important traits has already been demonstrated [24]. Agarwal et al. [25] also exploited the CTDB resource for identification of novel genic SSRs and confirmed their applicability in a select set of 44 chickpea genotypes. Thus, the present resource of microsatellites is expected to accelerate chickpea improvement programmes via molecular breeding approaches.

#### SNP search

A search option to facilitate the discovery of SNPs between different genotypes of chickpea has also been integrated in the CTDB (Fig 2). The CTDB contains 1986, 36446 and 37954 high-quality SNPs between kabuli/desi, desi/wild and kabuli/wild genotypes, respectively using data from previous studies [7,8]. This information can assist accessing diversity between various genotypes. The search system is also equipped with various parameters to make the retrieval system user-friendly, including a specific base change (e.g. A/T, G/A), read depth, location of SNP in the transcript and SNPs in the members of a given TF family. The use of these search parameters makes the data mining flexible and more relevant. The output also provides an option to download the downstream and upstream flanking sequences to the SNPs for designing PCR primers as per user requirement. This module will prove to be an effective tool to enable marker design for associated traits in chickpea crop improvement programs.

#### Comparative genomics analysis

An interface has been integrated in the database to conduct a comparative analysis of chickpea transcripts with other plant species, including legumes (*G*. *max*, *M*. *truncatula*, *L*. *japonicas*) and model plant Arabidopsis (Fig 2). This option provides several utilities, such as BLAST search, ortholog search and comparative expression search modules. The “BLAST Search” page offers opportunity to conduct similarity search for protein/nucleotide sequence(s) of chickpea against other legumes (*G*. *max*, *M*. *truncatula* and *L*. *japonicus*) and Arabidopsis. The putative orthologs of chickpea transcripts in legumes and Arabidopsis can be retrieved using “Ortholog Search” along with an option to download their corresponding protein sequences. Another search module, “Comparative Expression Search” has been provided to conduct comparative expression analysis of chickpea transcripts and their orthologs in legumes and Arabidopsis during different developmental stages. The CTDB data has been utilized for analyzing the influence of gene duplication and gene loss on evolution of heat shock transcription factor genes in legumes including chickpea [26]. This feature of CTDB will be a useful resource for the search of ortholog(s) and the comparative genomics analysis.

#### Download and help

The user can download the complete transcriptome of wild (PI 489777), kabuli (ICCV 2) and desi (ICC 4958) chickpea genotypes in fasta format. An additional tool, “Batch Download”, enables downloading of transcript data including the sequence, function, domain and GO for a given set of transcripts. In order to demonstrate the possible fundamental utilities of this database, we have contrived help option. This page provides tutorial and easy to follow step-wise instructions.

### Examples highlighting major applications of the database

**Fig 3:**
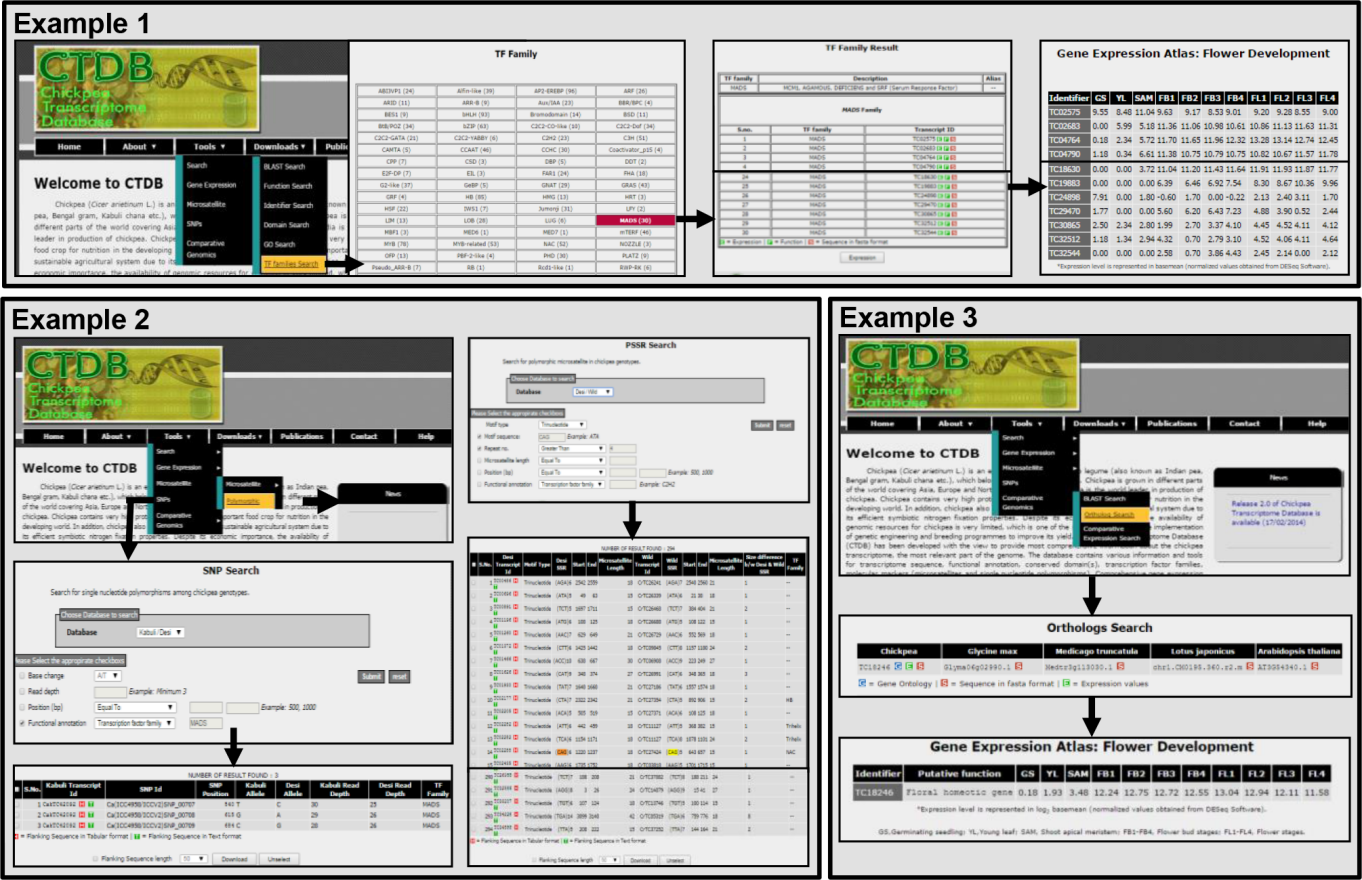
Screen shots of the CTDB demonstrating the use of CTDB database (v2.0) for various applications. The utility of CTDB have been demonstrated by presenting three examples. The retrieval of the expression profile of the transcripts encoding MADS-box transcription factor family members is the first example. Mining of polymorphic SSRs and SNPs between chickpea genotypes has been demonstrated in the second example. The identification of ortholog of a known gene in chickpea using comparative genomic approach is shown as third example.

To demonstrate the utility of the CTDB, we employed various features of the database for studying specific transcripts, discovery of polymorphic SSRs and SNPs lying in the transcripts and for performing comparative genomics analysis with other legumes and Arabidopsis (Fig 3).

#### Example 1. Analysis of gene expression patterns of MADS-box transcription factor gene family members

The expression analysis of candidate transcripts/genes during different tissues/developmental stages of chickpea is an important feature of the CTDB to facilitate their functional analysis. The demonstration of this utility has been presented by analyzing expression patterns of transcripts encoding MADS-box family transcription factors. Two steps are required to investigate the gene expression pattern of the transcripts of interest. In the first step, the CTDB provides a list of all TF families in chickpea with number of members under the “TF families search” tab. In the second step, a list of transcripts belonging to MADS-box family could be viewed by clicking on the hyperlink provided with name of MADS-box family in list of TF families. A hyperlink for each transcript ID, has been provided to access the respective expression profile during different tissues/developmental stages. An additional option at the bottom of table has also been provided to retrieve the expression profiles of all transcripts belonging to MADS-box family together during different tissues/developmental stages (Fig 3).

#### Example 2. Discovery of polymorphic SSRs and SNPs between desi and wild chickpea genotypes

This application has been presented with two examples to illustrate the utility of the CTDB in breeding approaches. The identification of polymorphic tri-nucleotide SSRs between desi and wild type genotypes is given as an example (Fig 3). A hyperlink of “Polymorphic” tab under “Microsatellite” enables screening of polymorphic SSRs with various search options. After selecting the “Desi and Wild” database, the customization of various parameters (motif type, trinucleotide; motif sequence, CAG; repeat number, greater than 4) can be carried out to retrieve the list of polymorphic SSRs of interest. The resulting page displays four polymorphic SSRs from desi transcript database with descriptions including their respective unique transcript identifier (TC02288, TC05305, TC05490 and TC06246), motif-type, repeat length, starting and end positions, length in desi and wild type and their related TF family (if any). The option to download the flanking sequence(s) of the selected SSRs of a particular length can be used to design PCR primers for genotyping applications.

The second example involves the identification of SNPs between desi and kabuli genotypes located within transcripts/genes of MADS-box family. The hyperlink of “SNPs” tab enables to access the search page for customization of the query. The selection of “Desi and Kabuli” as a database option along with relevant search options (function annotation; MADS), the result page displays three SNPs located in the CaKT42092 transcript. This page shows the position of SNPs within transcript with allele and read depth in desi and kabuli genotypes.

#### Example 3. Identification and characterization of an ortholog of a transcript of known function from *M. truncatula* in chickpea

An illustration has been presented to characterize an ortholog transcript in chickpea corresponding to *M*. *truncatula*. The AP3-like gene (*MtNMH7*) from *M*. *truncatula* (NCBI GenBank accession number JN412096.1 mRNA), which is predicted to belong to B-class MADS-box gene family was randomly selected as an example for identification of ortholog transcript in chickpea. The functional analysis of *MtNMH7* has shown its contribution in petal identity [27]. For identification of its ortholog in chickpea, the first step is to perform “BLAST search” of *MtNMH7* sequence in the chickpea transcripts. A transcript (TC18246) with putative function of floral homeotic gene showed highest similarity to the *MtNMH7* sequence. The TC18246 transcript was found to be involved in petal and stamen development via GO annotation using CTDB database. The expression of TC18246 transcript using “GEA: Flower Development” was found to be highest in various stages of flower development as compared to others tissues, as expected. This observation is in agreement with previous work in which the expression of *MtNMH7* have been exclusively detected in floral tissues of *M*. *truncatula* [27].

In addition to these demonstrated examples, CTDB will be useful for various genomics studies that will help to facilitate chickpea improvement programs.

### Future developments

The database will be updated regularly as and when the new data are available to expand utilities of CTDB. Additional features may also be included in the database to cater the demands of researchers in future.

### Availability and requirements

The CTDB 2.0 web service is freely available at http://nipgr.res.in/ctdb.html.

## Conclusions

In summary, the CTDB (v2.0) provides a user-friendly platform for many useful features/data to study the transcriptome of various chickpea genotypes via multiple search modules, expression analysis and comparative transcriptome analysis. The transcriptome datasets integrated in this database are potential source for the discovery of novel genes/transcripts in chickpea. In addition, this database presents the functional molecular markers (microsatellites and SNPs) to facilitate the molecular breeding programs. Altogether, this database is a significant advancement towards establishment of transcriptome resource for chickpea and is expected to accelerate functional and applied studies in chickpea and related legumes.

## Methods

### Database construction

The CTDB (v2.0) is presently hosted on a Sun Microsystem Workstation with two Intel Xeon quad core processors and 12 GB of random access memory which runs Linux operating system (Centos v5.5). MySQL relational database management system (v5.0.77) has been used to store and manage the processed transcriptome data [4]. The client web interface queries the Apache HTTP server (v5.5.29) to access the data from MySQL databases. Perl (v5.8.8), JavaScript (v1.6.0) and HTML (v4.0.1) based technologies have been employed to create the query-builder module for connecting user queries to the database.

### Data source, processing and implementation

The transcriptome datasets of wild [8], and kabuli [7] and desi [4] chickpea have been utilized for the construction of this database. The resources for the identification of putative function, similarity searching and prediction of transcripts encoding for transcript family have been utilized from previous study [4]. The functional molecular markers (microsatellites and SNPs) data have been utilized from previous studies [7,8] The normalized expression data for chickpea transcripts from major tissues (reads per kilobase per million) and various stages of flower development (log_2_ basemean value) were obtained from the previous studies [4,11] for integration into CTDB.

The genomic information for other legumes, *G*. *max*, *M*. *truncatula* and *L*. *japonicus* was retrieved from Phytozome v9.1 and Plant GDB databases. The Arabidopsis information resource (TAIR) was utilized to access genomic information of Arabidopsis. For comparative transcriptome analysis, orthologs of chickpea transcripts in other legumes and Arabidopsis were identified using reciprocal blast search (*E*-value ≤1e-5). The orthologs from different legumes and Arabidopsis were compiled and stored in MySQL. The normalized gene expression data of the selected tissues/organs for Arabidopis, *G*. *max*, *M*. *truncatula* and *L*. *japonicus* were retrieved from TAIR (ftp://ftp.arabidopsis.org/home/tair/Microarrays/analyzed_data/affy_data_1436_10132005.zip), Soybean Atlas [14], MtGEA [13] and LjGEA [18], respectively. These data were tabulated and stored in the MySQL database.
